# Attitudes towards breast conservation in patients aged over 70 with breast cancer

**DOI:** 10.1186/s40064-016-2133-5

**Published:** 2016-04-18

**Authors:** L. I. Smith, S. Dayal, J. Murray, A. Lannigan

**Affiliations:** Department of Breast Surgery, Wishaw General Hospital, 50 Netherton Street, Wishaw, ML2 0DP UK; Department of General Surgery, Basingstoke and North Hampshire Hospital, Aldermaston Road, Basingstoke, Hampshire RG24 9NA UK

**Keywords:** Breast cancer, Breast conserving surgery, Elderly

## Abstract

**Background:**

The majority of breast conserving surgery (BCS) is performed in younger women. There is little published information about the views of women aged over 70 regarding BCS. The aim of this study was to investigate the attitudes of this age group towards BCS, and factors which may influence their treatment decision-making.

**Methods:**

A questionnaire was sent to all patients who were aged 70 or over at the time they had breast cancer surgery in NHS Lanarkshire between 1999 and 2013. This detailed surgical options and recommendations, timing of decision making, treatment expectations, psychological and cosmetic concerns and other factors which may have influenced any decision made e.g. travel for radiotherapy and potential side effects.

**Results:**

Responses were received from 339 patients, 192 of whom had a mastectomy with the remaining undergoing BCS. In the mastectomy group 18 % (35) would have preferred to have BCS had it been an option, with 40 % (76) of group being happy to take neoadjuvant endocrine therapy to try and facilitate this. However, only 14 % (26) of patients would have considered neoadjuvant chemotherapy with the same aim. Almost half (82) of the mastectomy patients said that the risk of local recurrence following BCS was a factor which influenced their decision.

**Conclusion:**

BCS is something that patients aged over 70 are interested in considering in the same way as younger patients. More than a third of patients requiring mastectomy would be willing to take neoadjuvant endocrine therapy to attempt to downstage their tumour to facilitate BCS.

## Background

At the current time 24 % of all breast cancer cases diagnosed in the United Kingdom are in women over the age of 75, and it is well established that that the risk of developing breast cancer increases with age (Cancer Research UK 2015). The number of new cases diagnosed in older women is predicted to rise by a factor of four over the next twenty-five years (Maddams et al. 2012).

Elderly patients differ in a number of important respects from younger women and these may affect the outcome of their breast cancer treatment. They have increased co-morbidities in addition to different psychological and functional profiles (physical capabilities and activities of daily living; Yancik et al. 2001). They are also less likely than younger women to ask questions or to seek a second opinion (Wyld and Reed 2003).

Various guidelines exist with regard to the treatment of older patients with breast cancer. These recommendations tend to be based on extrapolated results from younger patients, or small retrospective sub-group analysis due to lack of level 1 evidence in the older population as historically they have been excluded from randomised controlled trials (Biganzoli et al. 2012). This is despite studies demonstrating that the elderly are willing to participate in breast conservation trials (Silliman et al. 1993).

National Institute Clinical Excellence (NICE) guidelines on the diagnosis and treatment of locally advanced breast cancer (National Institute for Health and Care Excellence guidance 2009) state: “treat patients with early and locally invasive breast cancer, irrespective of age, with surgery and appropriate systemic therapy, rather than endocrine therapy alone, unless significant co-morbidity precludes surgery”. This is mirrored by the updated (2012) International Society of Geriatric Oncology (SIOG) and European Society of Breast Cancer Specialists (EUSOMA) recommendations on the management of older women with breast cancer (Biganzoli et al. 2012). These state that “patients 70 years or older should be offered the same surgery as younger patients”, they go onto specify the standard of care to be breast conserving surgery (BCS) plus whole breast radiotherapy (WBRT) or mastectomy with or without radiotherapy. Indications for mastectomy are listed as large, or multifocal tumours not amenable to BCS, patients not fit for WBRT, and most notably patients who prefer mastectomy to BCS and WBRT.

Even though age alone is not a contraindication for BCS (Morrow et al. 1998) several retrospective studies have demonstrated that elderly women are less likely to be offered this option, with Repetto et al. showing that BCS was undertaken in 71.1 % of women under the age of 50, and in only 26.1 % of women ≥70 (Repetto et al. 1997). Furthermore treatment decisions are often based on arbitrary reasons rather than evidence-based medicine (Newschaffer et al. 1996).

Treatment of breast cancer by mastectomy or BCS are equally effective in terms of survival (Fentiman et al. 2003). When BCS is followed by radiotherapy, local recurrence rate is no different than that following mastectomy alone (Early Breast Cancer Trialists’ Collaborative Group 1995). There is also evidence that women undergoing BCS have a better quality of life than those who had mastectomy (Curran et al. 1998).

To extend the use of BCS, there has been increasing use of both neoadjuvant chemotherapy and endocrine therapy to reduce tumour size prior to surgery (Kurtz 1991; Fisher et al. 1998; Mauriac et al. 1991, 1999; Robertson et al. 1992; Semiglazov et al. 1994; Gazet et al. 1994; Mustacchi et al. 1994; Bates et al. 1991). Endocrine therapy is much better tolerated than chemotherapy which is important in elderly patients who may have other significant co-morbidities. Tamoxifen was the first widely used neoadjuvant endocrine treatment and has been shown to be successful in elderly patients at reducing tumour size (Keen et al. 1997). This allowed inoperable tumours to become operable and tumours which would have required mastectomy to be removed by wide local excision (Gazet et al. 1994; Mustacchi et al. 1994; Bates et al. 1991; Van Dalsen and de Vries 1995). More recent studies suggest that Letrozole is an even more effective agent in this regard in postmenopausal women with a higher response rate to treatment and more patients being able to have breast conservation (Eiermann et al. 2001).

Factors implicated in the decision making process for the treatment of breast cancer have been reviewed in the literature. Advice given by the surgeon (Rippy et al. 2014) and age being quoted as important factors (Sio et al. 2014). However, there is little specific to the elderly beyond one study reviewing the over 80s, which showed the risk/benefit profile of treatment, logistics (including transport), and psychosocial characteristics of the patients to be influential (Schonberg et al. 2012). Meanwhile across a range of ages body image, survival benefit, risk of recurrence and quality of life have been highlighted as significant factors in a recent systematic review (Hamelinck et al. 2014).

There is little information in the literature about the views of women aged over 70 regarding BCS and the factors which influence their decision about which type of surgery to choose (Sandison et al. 1996). Figueiredo investigated long-term body image and mental health in the elderly after breast cancer treatment, specifically considering whether patient preferences will predict post- treatment outcomes (Figueiredo et al. 2004). They concluded that body image is important for many older women and that considering their treatment preferences is important with regard to long-term mental health outcomes. Shared decision making should be a focus of treatment in older women.

The aim of this study was to investigate the attitudes towards BCS, and factors which may influence decision making in women over 70 with newly diagnosed breast cancer.

## Methods

A database of all patients diagnosed with breast cancer across the three hospitals in NHS Lanarkshire is prospectively maintained. From this patients aged 70 and over at the time of breast cancer diagnosis between 1999 and 2013 were identified. Those who were treated operatively (mastectomy or BCS) were sent a questionnaire through the postal service (“Appendix”) covering potential surgical options and recommendations, timing of decisions made, treatment expectations, psychological and cosmetic concerns and other factors which may have influenced decision making e.g. travel for radiotherapy and potential side effects. The questionnaires were sent in two data sets, the first containing patients between 1999 and 2005, the second from 2006 to 2013. Responses were collected in 2005 and 2013 respectively and were entirely anonymous. The aim of the questionnaire was to ascertain why these patients chose to have their particular operation and what factors influenced their decision.

Patients excluded were men, those who had opted for primary endocrine treatment, patients unsuitable for operative treatment (e.g. advanced metastatic disease, inoperable tumours, significant co-morbidites precluding general anaesthetic), and death since diagnosis.

Patients were asked to respond with “yes” or “no” to questions such as “did the surgeon explain the different surgical options for treating your breast cancer?”. A scale was provided with the options “very worried”, “fairly worried”, “slightly worried”, “not worried” or “indifferent” for questions relating to the psychological or cosmetic effects of their surgery. For questions relating to post operative appearance of their breasts the options provided were “very unhappy”, “unhappy”, “unsure”, “happy” and “very happy”. Outcome measures for factors associated with decision making were chosen based on those published in the literature (Rippy et al. 2014; Sio et al. 2014; Schonberg et al. 2012; Hamelinck et al. 2014) in addition to the results of a small pilot group which were surveyed prior to the study being commenced.

Questionnaires were analysed in two groups, mastectomy vs. BCS. Statistical analysis was performed using Chi squared for comparison categorical proportions between the two groups. Students *T* test was utilised for comparing the continuous data related to tumour size. GraphPad Software (GraphPad software, Inc) was used for the calculations.

Ethical approval was sought and obtained from the local research ethics committee (LREC 06/S1001/10) and the NHS Trust research and development committee prior to the commencement of this project.

## Results

Five hundred and thirty-two patients were identified as being 70 years of age or older at the time of their diagnosis and subsequent operative management for breast cancer, all of whom were sent questionnaire. The mean age of women in both the BCS and mastectomy groups was 76 years. Median ages were also comparable, interestingly being younger in the mastectomy group (75 years, range 70–100) than the BCS (76 years, range 70–94). Total tumour size (including ductal carcinoma in situ) varied between the two groups with a mean of 20.4 mm in the BCS group, and 29.6 mm in the mastectomy group (p ≤ 0.00001).

Responses were received from 339 patients (64 %) and, of these, 192 patients had a mastectomy (57 %) and 145 had BCS performed (43 %). In the remaining two cases the patients did not indicate what surgery they had so were excluded from the main analysis. Unfortunately, due to the anonymisation of responses we are unable to provide any further information pertaining to the non-responders group.

Table 1 summarises the key comparable findings between the two groups.

**Table 1:** Summary of comparable findings

	Mastectomy	BCS	p value
*Patient demographics*			
Mean age	76 years	76 years	Not significant
Median age	75 years	76 years	Not significant
Age range	70–100 years	70–94 years	
Total tumour size	29.6 mm	20.4 mm	p ≤ 0.00001
*Treatment recommedations*			
Underwent the recommended treatment	86 %	86 %	Not significant
Other treatments discussed	58 %	70 %	p = 0.02
More information requested	26 %	26 %	Not significant
*Treatment expectations*			
Patients expected a mastectomy at diagnosis	64 %	33 %	p = 0.0001
*Cosmetic and psychological concerns*			
Worried about the cosmetic effect of mastectomy	38 %	26 %	p = 0.013
Worried about the psychological effect of mastectomy	55 %	37 %	p = 0.0008

### Treatment recommendations

Of the patients who had a mastectomy, 86 % (162) said that their surgeon had recommended this as the best surgical option; this was identical in those who underwent BCS (86 %, 118 patients). However, fewer patients in the mastectomy group recalled their surgeon discussing different surgical treatment options than in the BCS group (58 %, 112 vs. 70 %, 102; p = 0.02). This may reflect the fact that BCS may not have been an option for those who had a mastectomy. Twenty-six percent of patients (49 in the mastectomy group and 38 in BCS group) in both groups requested more information about the surgical options.

### Treatment expectations

Patients in both groups expected that they would require mastectomy when first informed they had breast cancer. Interestingly, this was the case for significantly more patients (p = 0.0001) who had a mastectomy (64 %, 123 patients) than patients who had BCS (33 %, 48 patients). Of the patients who had a mastectomy, only 26 % (50) had discussed the possibility of BCS pre-operatively with their surgeon. Eighteen per cent (35) of mastectomy patients said they would have preferred BCS if that had been a treatment option, the majority being unsure (118 patients, 61 %).

### Factors influencing decision making

**Fig. 1:**
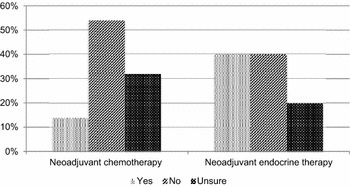
Numbers of women willing to take neoadjuvant treatment to facilitate BCS

Within the mastectomy group, 40 % (76 patients) said they would have been happy to take neoadjuvant endocrine therapy to shrink their tumour pre-operatively to facilitate BCS. However, only 14 % (26 patients) would have considered neoadjuvant chemotherapy with the same aim (Fig. 1).

**Fig. 2:**
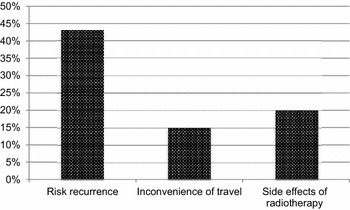
Factors affecting decision in favour of mastectomy

Forty-three percent (82) of all mastectomy patients said that the possibility of local recurrence following BCS was a factor which influenced their treatment decision. Only 15 % (28) of patients felt that having to travel for at least 2 h per day to attend for post-operative radiotherapy put them off having BCS. A fifth of patients (39) felt that radiotherapy might have significant enough side effects to put them off having BCS (Fig. 2).

Although those within the mastectomy group were concerned about the side effects of radiotherapy, of the 145 patients who had BCS, 40 (28 %) reported no side effects at all. The majority of those who had problems suffered only minor skin reddening or tiredness (52, 36 %). Twelve people (8 %) had a poor cosmetic effect from breast shrinking or asymmetry as a result, with 14 (10 %) suffering from more severe complications (lung, skin breakdown). Despite our patients having to travel a considerable distance daily to Glasgow for radiotherapy, only 4 % (6 patients) felt that this was a problem.

### Cosmetic and psychological concerns

**Fig. 3:**
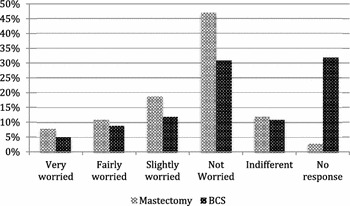
Cosmetic concerns associated with having a mastectomy

**Fig. 4:**
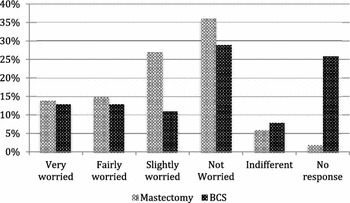
Psychological concerns associated with having a mastectomy

Interestingly, 38 % (74) of patients who opted to have a mastectomy and only 26 % (37) of patients undergoing BCS were worried about the cosmetic effects of losing a breast (very worried, fairly worried and slightly worried), this was not statistically significant (p = 0.013) (Fig. 3). Whereas concerns over the psychological consequences of mastectomy were significant (p = 0.0008) with 55 % (106) of mastectomy patients and 37 % (53) of BCS patients being worried that a mastectomy may have led to feelings of low self-esteem or depression (very worried, fairly worried and slightly worried; Fig. 4). It should also be noted that the level of non-response was much larger within the BCS group for both the cosmetic and psychological concerns (32 and 26 % respectively) when compared to the mastectomy group (3 and 2 %).

**Fig. 5:**
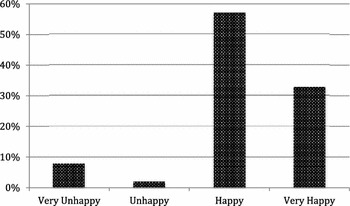
Satisfaction with cosmetic results of BCS

Of patients who had a wide local excision, 88 % (127) said they were happy with their decision to have BCS. Of these, 90 % (104) said they were happy or very happy with their cosmetic outcome. However, 8 % (9) of BCS patients reported being very unhappy with their cosmetic result (Fig. 5).

## Discussion

Breast cancer incidence increases with age and is becoming more common with the demographic shift to an older population. One-third of patients with breast cancer are aged 70 or over at the time of diagnosis (Ganz et al. 1992). Many elderly patients continue to enjoy excellent physical and cognitive health well into their eighth and ninth decades making it appropriate that they receive treatment that gives long-term disease control and is to their satisfaction.

### Treatment recommendations

It has been established that the use of primary endocrine therapy alone in patients who are fit for surgery and the reluctance to subject elderly patients to post-operative radiotherapy can no longer be justified (Horobin et al. 1991). There is no evidence that radiotherapy is less favourably tolerated by the elderly and, in fact there is evidence to the contrary (Dixon 1992). For this reason, mastectomy or wide local excision followed by post-operative radiotherapy is the optimal treatment modality in elderly patients who are fit for surgery (Yiangou et al. 1996; Morris et al. 1997), as confirmed by the NICE guidelines (National Institute for Health and Care Excellence guidance 2009) and recommendations from SIOG & EUSOMA (Biganzoli et al. 2012).

Eighty-six percent of out patients recalled being given a recommended treatment option by their surgeon, be it mastectomy or BCS, suggesting that our centres are complying with these guidelines. This is the case despite those within the mastectomy group being less likely to receive a choice of surgical options (p = 0.02), reflecting the lack of options within the group.

Elderly patients with breast cancer are less likely to be offered adjuvant chemotherapy than younger women for several reasons. They are perceived to be less likely to derive benefit from chemotherapy although most trials of chemotherapy exclude this age group. In addition increased co-morbidity leads to a greater risk of complications. This study indicates that only a few women (14 % of those undergoing mastectomy) would be prepared to consider neoadjuvant chemotherapy to downstage their tumour to facilitate BCS. Whereas the same patients are far more amenable to the use of neo-adjuvant endocrine therapy.

### Treatment expectations

To date there is little published in the literature about the treatment expectations of the elderly. We have shown that patients in both groups initially expected to have mastectomy, although this was the case for significantly more patients who subsequently had a mastectomy (p = 0.0001). This maybe the case as breast conservation is a relatively new treatment, and that our patients were drawing treatment expectations from the treatment of the previous generation.

### Factors influencing decision making

It is interesting to note that few studies have examined the issues surrounding patient choices about treatment in this age group. A small study (n = 50) of women aged over 70 with breast cancer which was published in 1996 concluded that if given a choice, this age group would choose BCS and post-operative radiotherapy as opposed to modified radical mastectomy (Sandison et al. 1996). However, two more studies reported that mastectomy was the more common choice for older women (Monypenny 2003; Wyld et al. 2004). This was considered to be because they presented with larger cancers which were unsuitable for BCS together with patient choice. The latter was thought to be due to the fact that elderly women were less concerned with body image and chose what they considered to be the more simple therapeutic option of mastectomy (Wyld et al. 2004). Older women may be particularly concerned about their ability to remain independent when making treatment decisions. Therefore, if given a choice, many may prefer to have surgical treatment that does not require additional procedures such as subsequent radiotherapy (Liang et al. 2002).

In this study, 40 % of the patients who had a mastectomy said that they would have considered neoadjuvant endocrine therapy to facilitate BCS if that had been an option (Fig. 1). This was not routinely discussed as a potential treatment option during the whole study period. It is possible that using neoadjuvant endocrine therapy more frequently would increase the rate of BCS performed in this age group; however, clearly there would be cases in which this would not be appropriate.

### Cosmetic and psychological concerns

This study identified a large proportion of elderly breast cancer patients that may be affected by psychological problems such as low self-esteem and depression, or worry about the cosmetic effects of losing a breast. This may be something that clinicians and breast care nurses need to focus on more when counselling elderly patients both before and after breast surgery. Women are better informed than ever about the various treatment options in breast cancer and alternatives to mastectomy. We have also demonstrated that BCS is something that women aged 70 and over are interested in discussing. It has been suggested that when asked retrospectively, patients tend to favour the treatment they have undergone because it is thought that if they were to prefer another treatment option over their own, this could lead to regret or concern in thinking that their treatment was less than optimal (de Haes et al. 2003). This may explain why a larger percentage of women (which was statistically significant) having a mastectomy (65 vs. 33 %) expected to need a mastectomy pre-operatively, even though we found 18 % of the mastectomy group who would have preferred to have BCS had it been an option. This was demonstrated to the point where 40 % would consider neoadjuvant endocrine treatment, and 14 % neoadjuvant chemotherapy to help downstage their tumour to facilitate BCS. This could well be as a result of recall bias influenced by body image and the experience of mastectomy, in which case it is interesting to note what effect a mastectomy can have on elderly women.

### Limitations

Attention must be drawn to the inherent weakness in this type of study. Given the retrospective data collection recall bias is an issue, which we have already mentioned. This could also be worsened in our patient group due to their advanced age and potential cognitive impairment. We have attempted to reduce this by collecting the 14 years of data in two discreet groups, so that no patient had to recall a decision made over a decade previously. Clearly though 7 years is still a significant period of time to recall details from an emotional diagnosis and subsequent treatment. Response bias may also have played a part, as those patients with extreme views towards their outcome (positive, or negative) may have been more inclined to respond than those who were content with their treatment outcome. In addition, as no clinical assessment of the tumours is kept within our database, particularly in terms of local invasion, we can make no assessment whether or not those patients in the mastectomy group were potential candidates for BCS or not. Clearly suitability for BCS will alter conversations had at the initial diagnosis, and this could shape the patients perspective and views towards mastectomy as no other options are discussed.

Our response rate of 64 % could also be improved upon, but given the anonymous nature of our questionnaires, there was no way to track individual responses and pursue the non-responders accordingly. Despite these weaknesses, this is still a large sample (339 patients) of elderly patients expressing their views on their operative management, something that has not been seen to this scale before in the literature, and has an increasing impact in today’s population of breast cancer patients.

## Conclusion

BCS is something that patients aged 70 and over are interested in discussing and considering in exactly the same way as their younger counterparts. In this study, more than a third of patients requiring mastectomy would have been willing to take neoadjuvant endocrine therapy to attempt to downstage their tumour to potentially facilitate BCS. Although a few patients were put off by the requirement for post-operative radiotherapy, the majority of patients did not consider this to be a problem.
